# A Case of Immunotherapy-Induced Colitis Complicated by Perforation and Treated with Infliximab Postoperatively

**DOI:** 10.1155/2019/9069354

**Published:** 2019-07-22

**Authors:** Lukas Delasos, Aakash Desai, Nerea Lopetegui Lia, Nikhila Kethireddy, Carolyn Ray

**Affiliations:** ^1^Department of Medicine, University of Connecticut, Farmington, CT, USA; ^2^Department of Medical Oncology, Smilow Cancer Hospital at St. Francis, Hartford, CT, USA

## Abstract

The advent of checkpoint inhibitor therapy in medical oncology has led to an increase in hospitalizations for immune-related adverse effects. Severe colitis has been reported in approximately 5% of patients treated with cytotoxic T-lymphocyte-associated antigen-4 (CTLA-4) inhibitors, such as ipilimumab. Standard management for those with severe colitis includes administration of systemic corticosteroids with the reservation of antitumor necrosis factor (anti-TNF) therapy, such as infliximab, if there has been no improvement. Rarely, immunotherapy-induced colitis can become life-threatening and result in bowel perforation requiring surgical intervention. Yet, there are no specific recommendations for medical management following colectomy in these situations. In cases of severe colitis from Crohn's disease, postoperative treatment with infliximab has been found to be safe when administered shortly after intestinal resection. However, there remains limited data to support administration of infliximab following bowel perforation due to immunotherapy-induced colitis. Our case illustrates management of a severe adverse reaction to checkpoint inhibitor therapy and the need to further evaluate the role of infliximab postoperatively in patients who develop colitis complicated by bowel perforation.

## 1. Introduction

Favorable objective response rates and progression free survival have made the use of immune checkpoint inhibitors more widespread throughout the past decade for the treatment of both solid and hematological malignancies. Most notable are monoclonal antibodies directed against cytotoxic T-lymphocyte-associated antigen-4 (CTLA-4), programmed cell death 1 (PD-1) receptor, and programmed cell death ligands (i.e., PD-L1 and PD-L2) [1]. By blocking the interaction between CTLA-4 with B7 costimulatory molecules (CD80/86) or PD-1 with PD-L1/PD-L2, the normally associated T cell-suppressive response is prevented thus allowing for T cell-mediated destruction of tumor cells [1]. Identifying these mechanisms of T cell regulatory function has led to the largely adopted idea that the immune system can overcome the inhibitory effects of cancer cells with therapy directed against these signals. In fact, there has been widespread attention directed towards the treatment of melanoma with immunotherapy based on promising outcomes observed in clinical trials. Ipilimumab, a fully human monoclonal antibody (IgG1) that blocks CTLA-4, has not only shown improved survivability when combined with other therapeutic agents, including vaccines, but also as monotherapy for patients with metastatic melanoma [2, 3]. Additionally, patients with advanced treatment-refractory melanoma on maintenance nivolumab, a PD-1 immune checkpoint inhibitor, have demonstrated similar objective response rates and long-term safety as compared to matching patient populations with different solid tumor types [4].

With the advent of checkpoint inhibitor therapy in medical oncology, there has been an increase in the number of patients hospitalized with immune-related adverse effects. Retrospective analysis of immune-related adverse events leading to emergency department visits has demonstrated multiple organs affected, including gastrointestinal, pulmonary, cardiac, dermatologic, hepatic, pancreatic, and renal involvement [5]. Of these adverse effects, gastrointestinal toxicities have remained amongst the highest reported with complaints of diarrhea being most frequent [6]. The frequency of colitis in medical literature, for example, has ranged from 8% to 27% with the incidence of diarrhea being as high as 54% in patients treated with CTLA-4 inhibitors [6]. Those receiving a combination of CTLA-4 inhibitors and PD-1 inhibitors are at even greater risk for colitis as well as developing more severe symptoms (i.e., grade 3/4 colitis) [7]. Standard management for those with severe colitis includes administration of systemic corticosteroids with the reservation of antitumor necrosis factor (anti-TNF) biotechnical drugs, such as infliximab, if there has been no improvement [8]. Rarely, colitis can result in bowel perforation requiring surgical intervention as described by Marthey et al. in which 13% of patients who developed anti-CTLA-4 enterocolitis underwent colectomy for perforation [9]. Interestingly, fatal immune checkpoint inhibitor toxicities are most commonly due to colitis (70%) with anti-CTLA-4 inhibitors as compared to pneumonitis (35%) and hepatitis (25%) with anti-PD-1/PD-L1 inhibitors [10]. Here, we describe a case of severe immunotherapy-induced colitis complicated by bowel perforation requiring emergent colectomy and subsequent treatment with infliximab after failed steroid therapy.

## 2. Case Presentation

A 72-year-old male was found to have advanced melanoma metastatic to the liver and lung. Results from his biopsy were negative for BRAF mutation. Thus, the patient was eligible for first-line immunotherapy and began treatment with ipilimumab and nivolumab.

The patient began his first cycle of therapy with a single dose of ipilimumab, 290 mg, and nivolumab, 97 mg. Two weeks later, prior to any additional immunotherapy, he developed hematochezia. Given concerns for grade 3 drug-induced colitis, he was hospitalized and started on intravenous methylprednisolone 80 mg daily in addition to maintenance intravenous fluids. Stool studies were negative for *Clostridium difficile* toxin, *Salmonella, Shigella,* Shiga toxins 1 and 2*, Campylobacter, Escherichia coli O157:H7*, ova, and parasites. Endoscopic evaluation and intervention were deferred given the improvement of his symptoms while on steroids. The patient was then transitioned to oral prednisone starting at 40 mg with a plan to taper over the next several weeks.

Following his discharge, it was felt that he would be unable to tolerate continuation of CTLA-4 inhibitor immunotherapy with ipilimumab due to the increased risk of recurrent colitis. He was planned to resume PD-1 inhibitor immunotherapy with nivolumab every three weeks. However, prior to initiating monotherapy with nivolumab, he suddenly developed four episodes of diarrhea with associated nausea and abdominal pain classified as a grade 2 toxicity. He was administered a one-time dose of intravenous methylprednisolone 100 mg as an outpatient and instructed to increase his home dose of prednisone to 80 mg while holding the next cycle of nivolumab. Despite this, his symptoms progressed with the development of large volume hematochezia accompanied by severe right upper quadrant abdominal pain.

**Figure 1:**
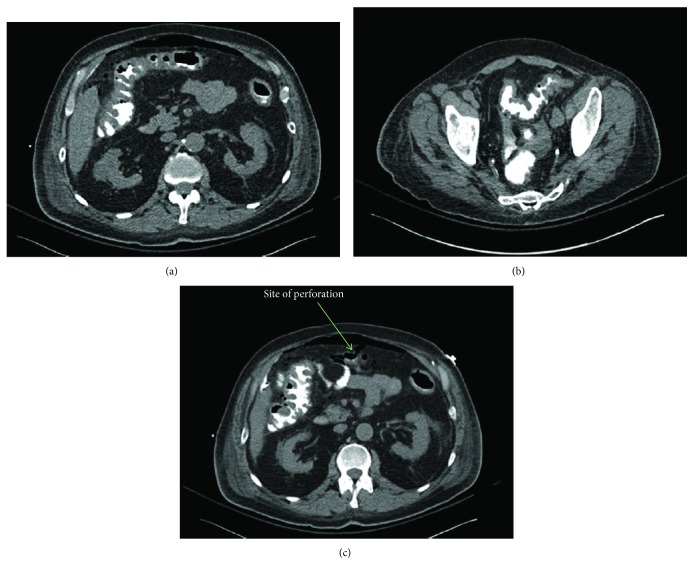


The patient immediately returned to the hospital at the direction of his oncologist. Stool studies were repeated and again found to be negative. Computed tomography (CT) imaging of the abdomen and pelvis revealed moderate circumferential thickening of the large bowel wall suggestive of pancolitis (Figures 1(a) and 1(b)). More importantly, there was evidence of pneumoperitoneum with a moderate amount of free air in the upper abdomen and a focal disruption noted in the anterior wall of the mid transverse colon which was felt to be the site of perforation (Figure 1(c)). Of note, the patient was also found to have had a significant response to immunotherapy with a decrease in size of his liver metastases by up to 70%. An emergent laparotomy was performed with extended right hemicolectomy and end ileostomy after perforations were identified along the cecum and transverse colon.

Postoperatively, he continued on intravenous steroids. However, on the first postoperative day, the patient had a large bloody bowel movement without evidence of blood in the ileostomy. His hematochezia continued into the second postoperative day. This raised concern for an increased risk of recurrent perforation given the patient's poor response to standard therapy and severe pancolitis evident on his presurgical CT scan. After a thorough discussion with both the surgical and gastroenterology teams, it was determined that the benefit of treating his ongoing colitis with infliximab outweighed the risks of adverse effects associated with this medication while in the postoperative state. He was administered one dose of infliximab 5 mg/kg intravenously with continued corticosteroid therapy. Over the next several days, the patient's symptoms improved without further episodes of rectal bleeding. Endoscopic evaluation was deferred by gastroenterology while the patient was hospitalized given evidence of clinical improvement and the high risk of iatrogenic bowel perforation. Shortly afterward, he was started on a clear liquid diet and gradually advanced to a low fiber diet with adequate function of his ileostomy. The patient's condition continued to improve, and he was eventually discharged to a rehabilitation facility with instructions to complete a slow taper of oral prednisone. Due to the severity of his immune checkpoint inhibitor toxicities, the patient was no longer a candidate for further immunotherapy. His performance status remained too poor to initiate second-line therapy, and surgical restoration of intestinal continuity was never performed.

## 3. Discussion

Immunotherapy-induced diarrhea and colitis remain the most frequent, serious, and potentially life-threatening adverse reactions to checkpoint inhibitor therapy. Grades of diarrhea and colitis are thoroughly described within the Common Terminology Criteria for Adverse Events (CTCAE v. 5.0). Anti-CTLA-4 agents have demonstrated a higher risk of inducing grade 3 or 4 diarrhea and colitis with rates up to 8.7% when used alone and as high as 13% when used in combination with anti-PD-1 agents [7]. However, anti-PD-1 agents such as nivolumab have been associated with a significantly lower incidence of both all-grade (2.9%) and grade 3 or 4 (1.8%) diarrhea and colitis when used as monotherapy [7]. Khoja et al. described patterns and incidence of immune-related adverse events based on cancer type and immunotherapy class in their large meta-analysis review. Within their findings, they describe a higher incidence of grade 3/4 adverse events when comparing anti-CTLA-4 inhibitors to anti-PD-1 inhibitors (31% versus 10%) [11]. Specifically, anti-CTLA-4 inhibitors have been identified as more frequent offenders for all-grade colitis, hypophysitis, and dermatitis whereas anti-PD-1 inhibitors were more commonly associated in cases of pneumonitis, hypothyroidism, arthralgia, and vitiligo [11]. In addition, they found that cases of melanoma treated with anti-PD-1 monoclonal antibodies were associated with a higher frequency of gastrointestinal and dermatological adverse effects as compared to other tumor types, namely non-small cell lung cancer and renal cell carcinoma [11].

**Figure 2:**
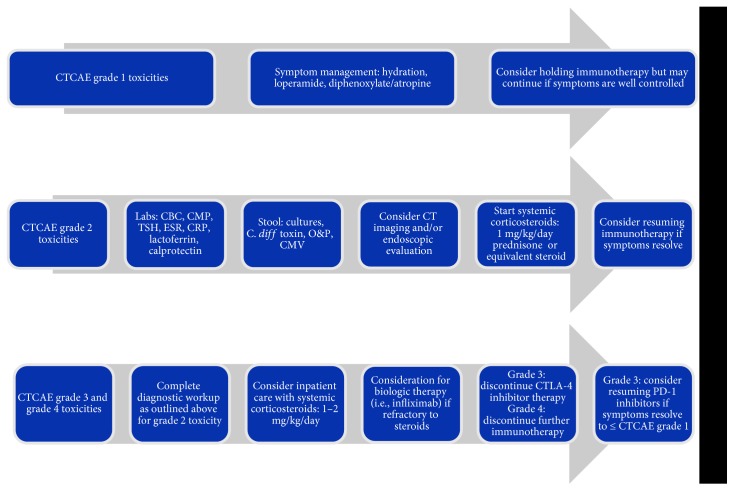
Recommended workflow for gastrointestinal toxicities. Abbreviations: CBC: complete blood count; CMP: complete metabolic panel; TSH: thyroid-stimulating hormone; ESR: erythrocyte sedimentation rate; CRP: C-reactive protein; *C. diff*: *Clostridium difficile*; O&P: ova and parasites; CT: computed tomography; CTLA-4: cytotoxic T-lymphocyte antigen-4; PD-1: programmed death-1.

Regarding management, the National Comprehensive Cancer Network (NCCN), American Society of Clinical Oncology (ASCO), and Society for Immunotherapy of Cancer (SITC) all recommend treating gastrointestinal toxicities based on the CTCAE grading system as noted above (Figure 2) [6, 12, 13]. For patients with grade 1 toxicities, immunotherapy may be temporarily held and resumed if symptoms do not progress [6]. Toxicities of grade 2 or higher severity require diagnostic workup including bloodwork (complete blood count (CBC), comprehensive metabolic panel (CMP), thyroid-stimulating hormone (TSH), erythrocyte sedimentation rate (ESR), C-reactive protein (CRP), and cytomegalovirus PCR); stool studies (cultures, *Clostridium difficile* toxin, ova and parasites, lactoferrin, and calprotectin); and consideration for both computed tomography imaging as well as endoscopic evaluation by gastroenterology [6, 13]. If grade 2 symptoms persist, providers should consider administering corticosteroid therapy. Higher grade toxicities usually require treatment with systemic corticosteroids at an initial dose of 1-2 mg/kg/day of prednisone or equivalent steroid, discontinuation of CTLA-4 inhibitors, and holding of PD-1 inhibitors until symptoms resolve to a grade 1 or less [6, 13]. Anti-TNF agents (i.e., infliximab, vedolizumab, and adalimumab) are usually reserved for cases refractory to management with corticosteroids.

The use of biologic agents to treat refractory cases of immunotherapy-induced colitis has proven to be effective at achieving remission. One prospective study showed rapid clinical improvement in four out of five patients treated with one dose of infliximab after failing standard therapy [14]. Retrospective analyses demonstrate statistically significant improvement in time to symptom resolution as well as shorter duration of steroid therapy in patients treated with infliximab and corticosteroids as opposed to those treated with corticosteroids alone [15]. In cases refractory to infliximab, NCCN guidelines recommend consideration of vedolizumab which has shown favorable outcomes and a good safety profile with significant clinical (86%), endoscopic (54%), and histological (29%) remission rates [16]. Because of these promising findings, there is now a motion towards earlier treatment with biotechnical agents based on endoscopic evaluation. Endoscopically visible ulcerations may be a surrogate marker for steroid-refractory disease as suggested by Wang and colleagues [17]. Usual histological features of acute colitis secondary to anti-CTLA-4 monoclonal antibodies include neutrophilic infiltration of the lamina propria and crypt abscess formation [9]. Patients with more severe endoscopic findings such as numerous ulcerations and/or pancolitis, while not necessarily correlating with the severity of diarrhea, are likely to require the use of biologic agents for remission [18]. This evidence argues for the necessity of early endoscopic evaluation and the practice of a top-down approach in the management of immunotherapy-induced colitis.

The association between CTLA-4 checkpoint inhibition and immune-mediated diseases has been described outside of the spectrum of oncology and immunotherapy. Heterozygous mutations in the CTLA-4 protein-encoding gene, *CTLA4*, causes hyperactivation of effector T cells in addition to loss of circulating B cells with an increase and accumulation of CD21 B cells in nonlymphoid organs [8]. Zeissig et al. identified in patients affected by early-onset Crohn's disease a novel missense mutation in *CTLA4* resulting in abnormal protein folding and structural stability that subsequently leads to severe systemic autoimmunity [19]. In addition to inflammatory bowel diseases, other immune-mediated inflammatory diseases such as rheumatoid arthritis, ankylosing spondylitis, and psoriasis are known to share a common pathological pathway involving an inappropriate release of inflammatory cytokines, particularly tumor necrosis factor (TNF) [20]. Important to note, Coutzac and colleagues analyzed TNF-alpha concentrations within colonic biopsies collected prior to steroid treatment in cases of immunotherapy-induced colitis and found significantly higher values of TNF-alpha within the mucosa of the biopsies collected from anti-CTLA-4-induced colitis than anti-PD-1-induced colitis [21]. TNF helps regulate multiple immune cell functions such as proliferation, differentiation, and apoptosis and is produced by various cell types including macrophages, NK cells, and T lymphocytes [20]. The dominant role played by TNF in immune-mediated inflammatory diseases explains why biologic agents targeted against TNF have been so effective in the management of these disorders [22]. For example, adalimumab, which was first approved for the treatment of rheumatoid arthritis, has proven efficacious in a wide range of other autoimmune disorders attributable to the shared pathogenesis of these diseases [23]. Inhibition of CTLA-4 by target-specific monoclonal antibodies such as tremilimumab, ticilimumab, and ipilimumab are believed to result in acquired autoimmune disease via these biological mechanisms. Similarities between the pathophysiology of both inherited and acquired inflammatory bowel disease help explain why anti-TNF agents are effective in treating immunotherapy-induced colitis. However, caution must be taken when considering these agents given their potential safety concerns including infections, immunogenicity, lymphomas, cardiac failure, and hepatotoxicity observed with long-term therapy [24].

Although there is adequate data supporting the use of anti-TNF agents in a wide variety of autoimmune disorders, there is little evidence on their efficacy and safety following surgical intervention for severe immunotherapy-induced colitis. The increased risk for fatal infections associated with these agents may give providers pause when considering their use. However, as with any intervention, one must weigh the risks and benefits to determine the best course of action for each case. In a randomized, controlled, multicenter study, Fukushima et al. found a significantly lower risk of disease recurrence in patients with Crohn's disease who had undergone intestinal resection for their disease when treated with infliximab (52.6% recurrence rate) as compared to those without infliximab therapy (94.7% recurrence rate) [25]. This study included 19 patients treated with intravenous infliximab 5 mg/kg immediately after surgery and on postoperative weeks 2, 4, and 6, which was then followed by continued therapy every 8 weeks for the next 2 years [25]. An additional randomized, prospective study evaluated disease recurrence in postsurgical patients with Crohn's disease treated with or without infliximab monotherapy every 8 weeks and found significantly improved clinical (93.3% versus 56.3%), serological (CRP < 0.3 mg/dL; 86.7% versus 37.5%), and endoscopic (78.6% versus 18.8%) remission rates in those treated with infliximab for up to 36 months [5]. Of note, there were no observable adverse events related to therapy within this study.

Our case represents a severe adverse reaction to checkpoint inhibitor therapy in which severe colitis refractory to standard therapy with systemic corticosteroids was complicated by bowel perforation. We also illustrate the efficacy of postsurgical treatment with infliximab in a patient with pancolitis at high risk for recurrent perforation. As the field of immunotherapy continues to evolve, so will the guidelines for management of immune-mediated toxicities. Therefore, it is necessary to further evaluate the role of infliximab and other biotechnical agents within this subset group of patients who undergo emergent surgical intervention for immunotherapy-induced colitis and who are at high risk for further complications related to the severity of their toxicity. Furthermore, given the compelling results from small studies done to date and the more pervasive use of immunotherapy in the field of oncology, it is feasible to design larger and more controlled studies that assess toxicities and clinical outcomes in patients who initially receive immunomodulator therapy versus systemic steroids for the treatment of immune-mediated adverse effects.
